# Tetra-side-by-side technique using a multi-hole self-expandable metal stent with a 5.9-Fr stent delivery system

**DOI:** 10.1055/a-2763-5597

**Published:** 2026-01-08

**Authors:** Takeshi Ogura, Jun Matsuno, Takafumi Kanadani, Junichi Nakamura, Hiroki Nishikawa

**Affiliations:** 1 Endoscopy Center, Osaka Medical and Pharmaceutical University Hospital, Osaka, Japan; 2 13010 2nd Department of Internal Medicine, Osaka Medical and Pharmaceutical University, Osaka, Japan

**Fig. 1:**
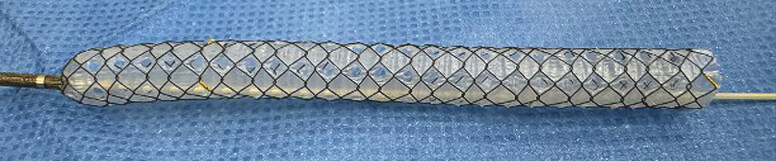
The multi-hole self-expandable metal stent with a 5.9-Fr stent delivery system (MHCSEMS; HANAROSTENT Biliary Multi-hole Benefit; M.I. Tech Co., Ltd, Pyeongtaek, South Korea).

Multiple stent deployment is sometimes necessary in cases of high-grade hepatic hilar obstruction. When uncovered self-expandable metal stent deployment is performed, the side-by-side (SBS) or stent-in-stent (SIS) technique can be selected 1 2 . However, stent patency might be limited due to the common complication of tumor ingrowth. Alternatively, a fully covered SEMS (FCSEMS) has the benefit of preventing tumor ingrowth, but it does not allow SIS and carries the risk of cystic duct or bile duct branch obstruction. A multi-hole self-expandable metal stent with a fine-gauge stent delivery system (MHCSEMS; HANAROSTENT Biliary Multi-hole Benefit; M.I. Tech Co., Ltd., Pyeongtaek, South Korea) that overcomes these limitations has become available ( Fig. 1 ). The stent prevents stent migration via small tissue ingrowth that occurs in the multiple small (1.8-mm) side holes along the covering membrane. The side holes also act to prevent cystic duct or bile branch obstruction. Because the stent delivery system is only 5.9 Fr, the system can be advanced easily and smoothly. Here, we report technical tips for the tetra-SBS technique using an MHSEMS for hilar obstruction.

**Fig. 2:**
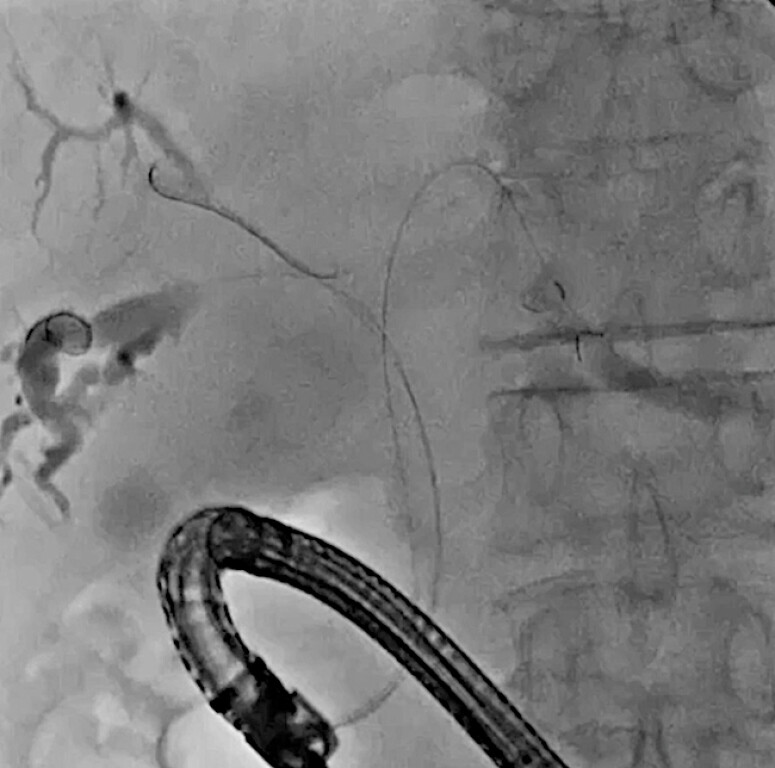
Guidewires are deployed into the left, anterior, and posterior bile ducts.

**Fig. 3:**
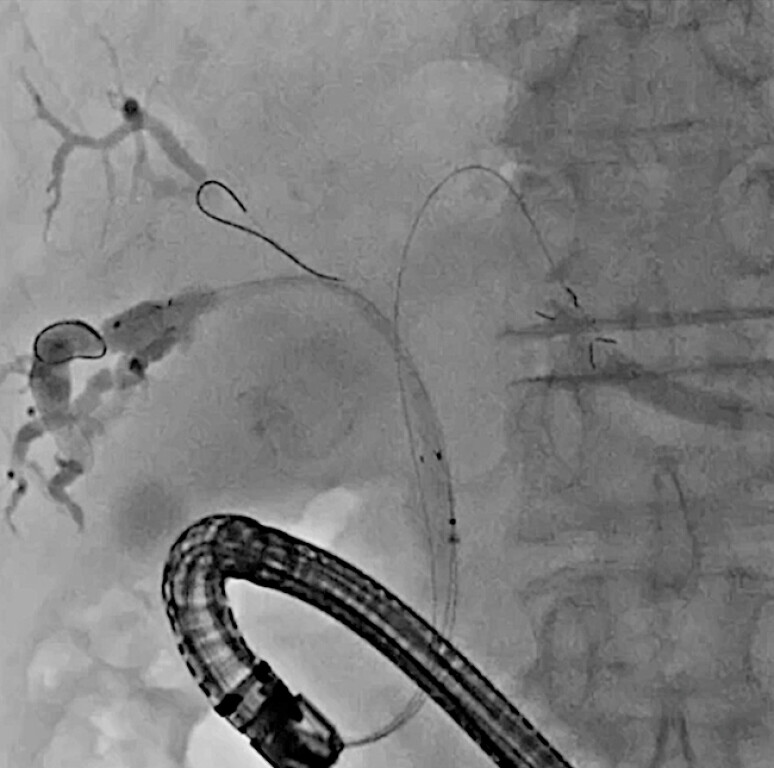
The MHCSEMS delivery system is inserted and deployed successfully. MHCSEMS, multi-hole self-expandable metal stent.

**Fig. 4:**
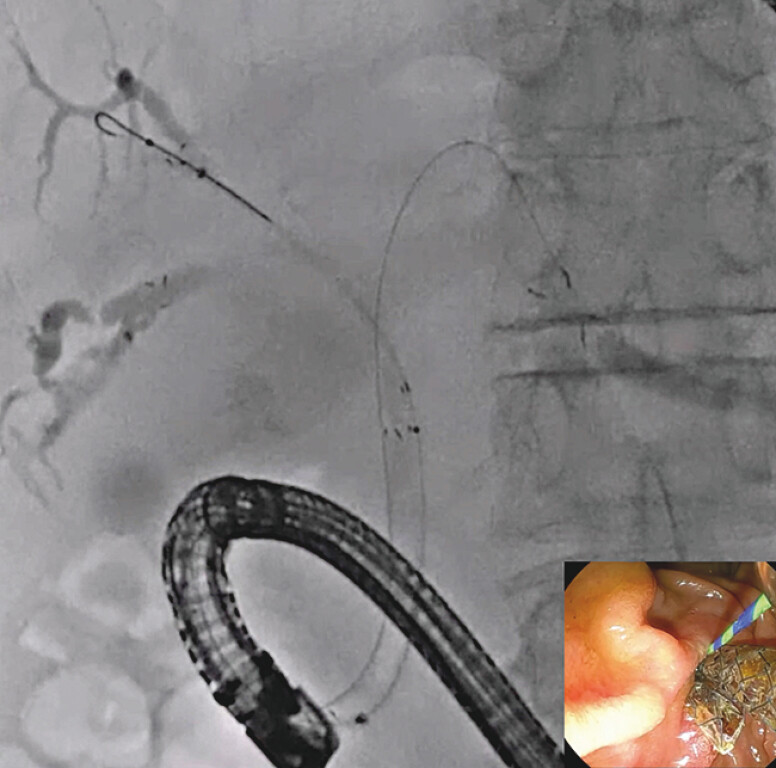
Successful stent deployment using an MHCSEMS for the anterior bile duct. MHCSEMS, multi-hole self-expandable metal stent.

**Fig. 5:**
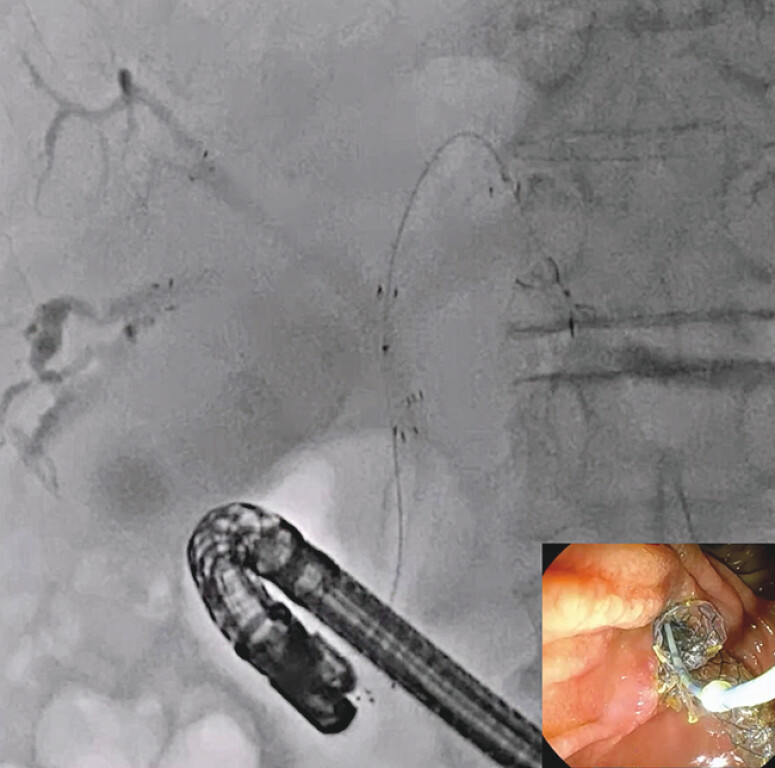
Successful stent deployment using an MHCSEMS for the left bile duct. MHCSEMS, multi-hole self-expandable metal stent.

A 77-year-old man underwent plastic stent deployment in the anterior and posterior bile ducts, and antegrade FCSEMS deployment in the left hepatic bile duct with hepaticogastrostomy following an unsuccessful left hepatic bile duct approach under endoscopic retrograde cholangiopancreatography guidance. Due to stent obstruction, endoscopic revision was attempted. First, the right-sided plastic stents and the FCSEMS were removed, and guidewires were deployed into the left, anterior, and posterior bile ducts ( Fig. 2 ). Hilar obstruction was observed on cholangiography. A stent delivery system was successfully inserted into the posterior bile duct ( Fig. 3 ), followed by successful stent deployment into the anterior bile duct ( Fig. 4 ). The fine-gauge stent delivery system was easily inserted into the left bile duct ( Fig. 5 ; Video 1 ) without any adverse events.

Tetra-side-by-side technique using a multi-hole self-expandable metal stent with a 5.9-Fr stent delivery system is performed.Video 1

In conclusion, the tetra-SBS technique using an MHCSEMS with a fine-gauge stent delivery system appears suitable for the insertion of a stent delivery system and prevents cystic or bile duct branch obstruction.

Endoscopy_UCTN_Code_TTT_1AR_2AZ
